# Unveiling Distinct Developmental and Cellular Impacts of Cisplatin on Zebrafish (*Danio rerio*) Embryos: Application of a Combined Developmental Toxicity with Rapid DNA-Content Flow Cytometry Protocol

**DOI:** 10.3390/mps9050132

**Published:** 2026-09-07

**Authors:** Maximos I. Leonardos, Vasileia Karavida, Anna Tsoukaneli, Dimitrios Leonardos, Yannis V. Simos, Konstantinos I. Tsamis, Dimitrios Peschos, Georgios S. Markopoulos, Lampros Lakkas

**Affiliations:** 1 Department of Physiology, Faculty of Medicine, School of Health Sciences, University of Ioannina, 45110 Ioannina, Greece; m.leonardos@uoi.gr (M.I.L.); karavidavicky@gmail.com (V.K.); a.tsoukaneli@uoi.gr (A.T.); isimos@uoi.gr (Y.V.S.); ktsamis@uoi.gr (K.I.T.); dpeschos@uoi.gr (D.P.); 2 Laboratory of Zoology, Department of Biological Applications and Technology, School of Health Sciences, University of Ioannina, 45110 Ioannina, Greece; 3 Department of Haematology, University Hospital of Ioannina, 45500 Ioannina, Greece; d.leonardos@uoi.gr; 4 Nanomedicine and Nanobiotechnology Research Group, University of Ioannina, 45110 Ioannina, Greece

**Keywords:** zebrafish, *Danio rerio*, cisplatin, flow cytometry, DNA-content analysis, developmental toxicology

## Abstract

Rapid cellular assessment of cell-cycle perturbation can complement conventional developmental toxicology in zebrafish, but rapid DNA-content workflows developed for mammalian tissues require adaptation for whole embryos. Here, we applied an embryo-adapted version of rapid DNA-content flow cytometry analysis within a 96-h cisplatin exposure model in zebrafish (*Danio rerio*) and integrated this cellular readout with morphometric, cardiac, and neurobehavioral analysis. Dechorionated embryos were exposed to 10–400 μM cisplatin and evaluated for body length, eye surface area, heart rate, locomotor activity, thigmotaxis, touch-evoked and vibrational startle responses, and whole-embryo cell-cycle distribution. The adapted workflow combines immediate mechanical dissociation, filtration, brief propidium iodide/RNase staining, singlet gating, and direct flow cytometric acquisition. Importantly, each flow cytometry sample consisted of a single embryo, enabling DNA-content analysis at the individual-embryo level without pooling. Cisplatin produced time- and concentration-dependent growth effects and high-dose behavioral alterations, whereas heart rate was comparatively preserved. Flow cytometry detected the clearest cell-cycle redistribution at 400 μM, with a significant reduction in G0/G1 and an increase in G2/M. These findings demonstrate the practical applicability of a combined rapid embryo-adapted DNA-content workflow as a complementary cellular endpoint in a zebrafish developmental-toxicity platform and support further methodological application across experimental settings.

## 1. Introduction

Platinum-based drugs represent one of the most effective and extensively employed classes of chemotherapeutic agents for the treatment of solid tumors. Cisplatin [PtCl_2_(NH_3_)_2_], the first platinum-containing anticancer drug approved for clinical use, remains a fundamental component of therapeutic protocols for malignancies such as bladder, lung, and head and neck cancers [1,2,3]. Its antineoplastic activity is primarily attributed to the formation of DNA cross-links, which interfere with DNA replication and transcription, ultimately triggering apoptotic cell death in rapidly dividing cancer cells [4]. Despite its proven clinical efficacy, the therapeutic application of cisplatin is frequently limited by dose-dependent toxicities, including nephrotoxicity, ototoxicity, and severe gastrointestinal complications [5]. Furthermore, the development of acquired chemoresistance significantly reduces treatment effectiveness, prompting ongoing efforts to optimize platinum-based therapies through the development of novel analogs and combination treatment strategies remains a major limitation of cisplatin therapy [6].

The extensive use of zebrafish in the scientific community is attributed to a multitude of advantages. External fertilization and the rapid ex utero development of embryos, which remain transparent during the early life stages [7], allow for continuous real-time microscopic observation of organogenesis and high-throughput phenotypic profiling [8,9]. Furthermore, the species’ high fecundity, with females producing hundreds of eggs every few days, ensures the statistical robustness of experimental data [10].

At the genetic level, complete genome mapping has revealed a 70% similarity with the human genome, having at least one obvious ortholog in relation to 80% of human genes with mouse orthologs and are widely used for disease modelling and pharmacological screening [11,12], supporting translational applications [13] and rendering it an exceptionally reliable tool for modeling human diseases and investigating the toxicity of pharmaceutical compounds, such as cisplatin [14]. The zebrafish and humans share many similarities in bioprocesses and genomes. The zebrafish has emerged as an ideal animal model suitable for behavioral studies due to the capability to study its larval locomotor activity [14]. Finally, the use of zebrafish in research can support the 3R principles (Replacement, Reduction, and Refinement) [15].

The present study had two linked aims: (i) to investigate the toxicological impact of the anticancer agent cisplatin on zebrafish embryos. The experimental approach involved exposing the embryos to the substance during critical developmental stages to evaluate mortality rates, alterations in heart rate frequency, and shifts in morphological and behavioral characteristics. (ii) to evaluate the practical application of an embryo-adapted version of a rapid DNA-content flow cytometry workflow as a complementary cellular endpoint within the same experimental platform. Through systematic recording and analysis, this research aims to contribute to a more profound understanding of the mechanisms of action and the adverse developmental effects associated with compound exposure, having cisplatin as a standard, in a vertebrate model [16].

## 2. Materials and Methods

### 2.1. Zebrafish Husbandry and Embryo Production

Adult wild-type (AB strain) zebrafish were maintained in a Zebtec housing unit (Active Blue Tecniplast, Milan, Italy) at 28 ± 1 °C, a pH of 6.5–7.5, and a conductivity of 500 ± 50 μS cm^−1^ under a 14:10 h light/dark cycle. Embryos were obtained by pairing adults (3:2 male:female ratio) in an iSpawn-S breeding tank (Tecniplast, Milan, Italy). Collected eggs were transferred to Petri dishes containing system water for subsequent screening.

### 2.2. Embryo Selection and Cisplatin Exposure

For toxicity assessments, cisplatin [PtCl_2_(NH_3_)_2_] working solutions (10–400 μM) were prepared by serially diluting a 1 mg/mL stock solution with system water. Healthy zebrafish embryos were selected by screening out unfertilized or developmentally abnormal eggs, followed by manual dechorionation at 24 h post-fertilization (hpf). Exposures were conducted in 24-well plates, with each well containing three embryos in 1.5 mL of the respective solution, yielding a total of 18 embryos per treatment group to ensure statistical robustness. A control group was maintained under identical conditions using system water.

The experimental organisms studied for morphological, functional and behavioral characteristics were randomly selected from the population of control and cisplatin-exposed animals, and their selection was completely blind.

### 2.3. Morphometric Measurements

#### Somatometric Analysis

To evaluate the developmental toxicity of cisplatin, the larval body length and eye surface area were quantified across all experimental concentrations and time intervals. Throughout the 96-h exposure period, larvae were monitored, photographed, and recorded using a stereo microscope (Olympus SZ61, Olympus, Tokyo, Japan) every 24 h. Body length was defined as the total distance from the anterior tip of the snout to the posterior tip of the caudal fin. For each experimental condition (concentration and time point), measurements were obtained from a minimum of 10 larvae, and all procedures were conducted in triplicate to ensure reproducibility.

### 2.4. Evaluation of Cisplatin-Induced Cardiac Development and Function

The effect of cisplatin on heart rate of zebrafish embryos as a function of its concentration and hours of exposure was studied to evaluate the effect on developmental ontogeny. 112–120 embryos per concentration were separately analyzed under a stereo microscope (Olympus SZX7 Stereo, Olympus, Tokyo, Japan, Olympus KL300 LED light, Olympus, Tokyo, Japan) and high-quality video recordings were obtained for 1 min using a Basler MED Ace camera and Basler Microscopy software (Basler MED Ace 2.3 MP 164 color, Basler Microscopy, Ahrensburg, GermanySoftware V2.1). The video recording frame rate was set to 25 fps. To eliminate any effect of temperature, the measurements were taken in a temperature-controlled room (28 °C) and the video recordings were performed using transmitted cold lighting (LED). The acclimatization period was set at 10 min before each recording. The heart rate of embryos, under the effect of various concentrations of cisplatin investigated on the first, second, third, and fourth day (24, 48, 72, and 96 h of exposure, respectively) after the start of the exposure. Moreover, particular care was taken, since an irregular heartbeat should not be recorded as being lethal [17].

### 2.5. Behavioral and Neurobehavioral Profiling

To evaluate the neurobehavioral impacts of cisplatin exposure, larval activity was recorded using EthoVision v.18 software (Noldus Information Technology, Wageningen, The Netherlands). Larvae were individually placed in 24-well plates (n=12 per treatment group) to assess sensory-motor and anxiety-like responses. Basal locomotor activity (BLA) and the visual motor response (VMR) were evaluated by tracking total distance traveled and mean velocity during alternating light-dark cycles. Thigmotaxis served as an index of anxiety, refers to the behavioral tendency of an organism to avoid open areas and remain close to walls (peripheral preference). Thigmotactic behavior and general motility of zebrafish (*Danio rerio*) larvae exposed to varying concentrations of cisplatin (control, 10 μM, 25 μM, 50 μM, 100 μM, 200 μM, and 400 μM) were evaluated using the DanioVision observation chamber equipped with EthoVision XT tracking software v.18(Noldus Information Technology, Wageningen, The Netherlands)). To quantify spatial preference and movement dynamics, each well was digitally divided into two regions: Arena: The total surface area of the well and Inner zone: A concentric center zone defined by a radius equal to half of the total well radius. Behavioral tracking parameters recorded per zone included total time (s), moved distance (mm), and mean velocity (mm/s).

To examine reflexive sensory-motor integrity, the touch-evoked motor response (TMR) was quantified following a light tactile stimulus to the rostral region via a fine glass capillary. Additionally, the vibrational startle response (VSR) was evaluated using a 10-s period prior to stimulus delivery (baseline/before) and for 10 s immediately following exposure to low-intensity (low tap, 1×) and high-intensity (high tap, 4×) mechanical tap stimuli.

### 2.6. Embryo-Adapted Rapid Flow Cytometric DNA-Content Analysis

Cell-cycle analysis was used to evaluate whether the embryo-adapted workflow could capture cisplatin-related changes in DNA-content distribution in zebrafish (*Danio rerio*) embryos. The procedure was based on an embryo-adapted version of the rapid Ioannina Protocol [18]. Dechorionated embryos at 24 hpf were exposed to increasing concentrations of cisplatin for 96 h. At the end of the exposure period, embryos from control and treated groups were collected and processed immediately for flow cytometric analysis. Each flow cytometry sample consisted of one embryo; embryos were not pooled. In each experimental series, three individual embryos were analyzed separately per condition. The complete flow cytometry experiment was independently repeated in three separate experimental series. The adaptation retained the rapid direct DNA-content analysis workflow while replacing tissue processing with whole-embryo mechanical dissociation.

Briefly, embryos were washed with phosphate-buffered saline (PBS) and mechanically dissociated to obtain single-cell suspensions. The suspensions were gently homogenized and filtered in order to remove tissue remnants and aggregates while preserving cellular and nuclear integrity, in accordance with the rationale of the published Ioannina Protocol.

For DNA-content analysis, cells were stained immediately with PI/RNase A staining solution to a final propidium iodide concentration of 125 mM and incubated for 3 min in the dark. Samples were then analyzed directly by flow cytometry without delayed processing.

Flow cytometric acquisition was performed under standardized instrument settings using 488 nm excitation and a PI-compatible emission channel. A total of 10,000 events were acquired per sample using a BD Accuri Plus flow cytometer (Waters Biosciences, Milford, USA). Debris and doublets were excluded by appropriate gating, and only singlet cells were included in the final analysis. DNA histograms were generated and used to determine the percentages of cells in G0/G1, S, and G2/M phases.

Cell-cycle distribution in cisplatin-treated embryos was compared with that of the control group in order to assess dose-dependent alterations in proliferative activity.

Results were expressed as mean ± standard deviation (SD), and statistical significance was set at *p* < 0.05. For each cell-cycle phase, differences among cisplatin concentrations were first evaluated by one-way ANOVA, followed by post hoc comparisons of each treatment group versus control using multiple-comparison correction.

### 2.7. Statistical Analysis

Data regarding body length, eye surface area, and heart rate were initially processed with the aim of Danioscope software (version 1.2, Noldus, Wageningen, The Netherlands) and subsequently analyzed using IBM SPSS Statistics (v. 31.0). The impact of cisplatin on morphometric and functional parameters was evaluated using a general linear model (GLM), with concentration and time (hours of exposure-hoe) as independent variables and body length, eye surface area, or heart rate as dependent variables.

Larval locomotor activity (distance and velocity) and external stimulus responses were quantified using EthoVision XT (v. 18, Noldus Information Technology, Wageningen, The Netherlands). Light-dark transition and startle parameters were further analyzed via GLM, using concentration as the independent variable and time as a covariate. Statistical significance was set at *p* < 0.05. Post-hoc multiple comparisons between concentration groups were performed using Tukey’s HSD and Bonferroni correction tests (a = 0.05).

Τo ensure the normality of the data distributions, the values were log-transformed and then the GLM analysis was used.

### 2.8. Ethics Statement

All experimental protocols in this study were approved by the Research Committee of the University of Ioannina and the Veterinary Department of the Region of Epirus (11044-29/7/2022). The protocols were in accordance with the Guide for the Care and Use of Laboratory Animals [19].

## 3. Results

### 3.1. Developmental Toxicity of Cisplatin

Embryonic exposure to cisplatin was evaluated across a concentration gradient (10–400 μM) up to 96 h of exposure. Key growth and physiological markers, such as body length, eye surface area, and cardiac rhythm, were monitored through daily imaging. Following the treatment period, we performed neurobehavioral testing (light-dark transitions and stimulus response) and utilized flow cytometry at the end of the exposure period to assess cellular DNA content.

### 3.2. Deformity Assessment

Following cisplatin exposure, zebrafish embryos were microscopically evaluated for major developmental abnormalities, including yolk sac and pericardial edema, spinal deformities, blood cell aggregation, and jaw malformations. No morphological deformities were observed in any of the assessed parameters.

### 3.3. Morphometric Changes in Body Length and Eye Surface

**Figure 1:**
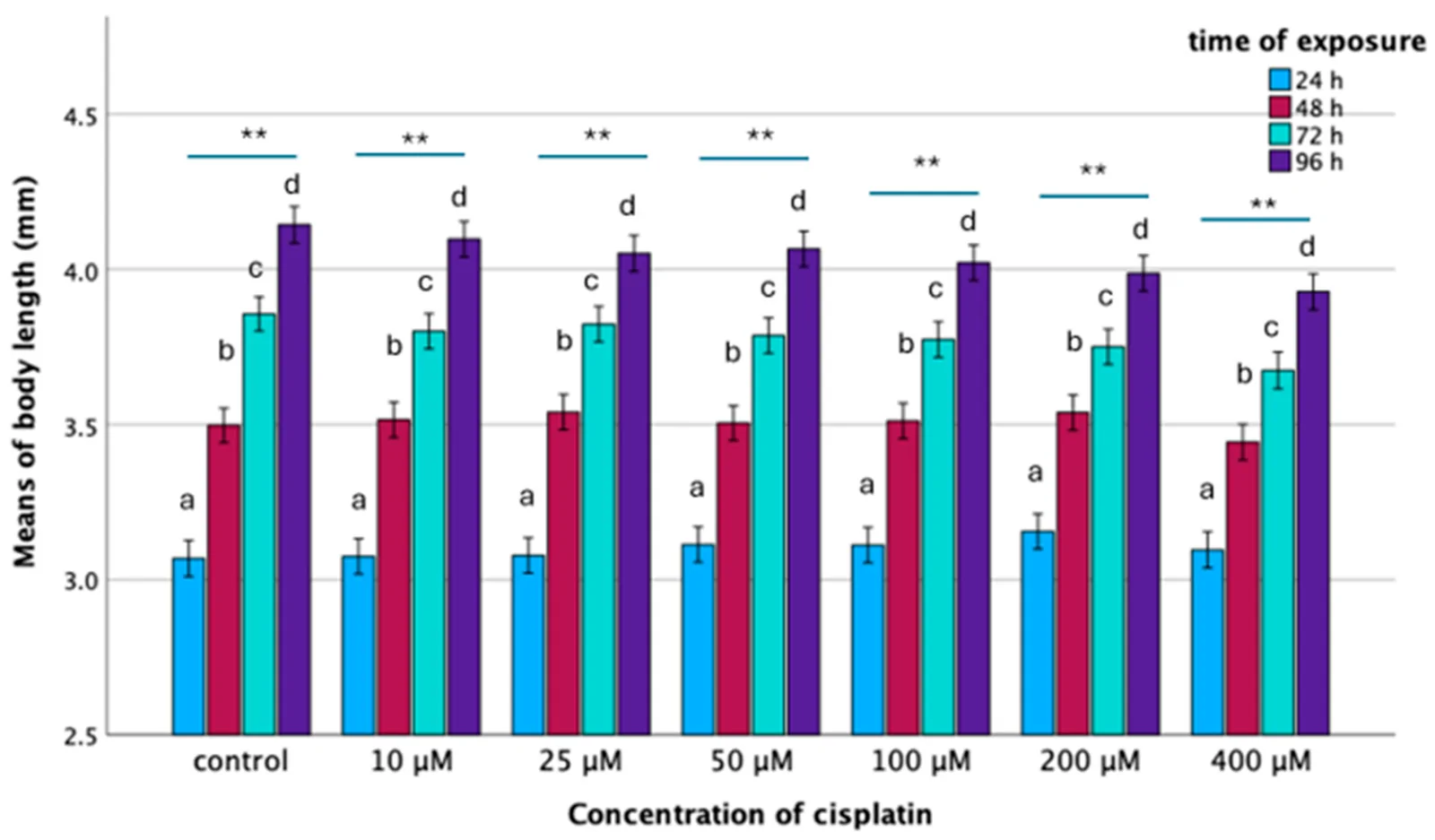
Mean body length (mm) of zebrafish embryos exposed to various concentrations of cisplatin per time points (hours of exposure). Horizontal bars above the groups show the comparison within each concentration in relation to different time points. Asterisks denote the level of significance (** indicates *p* < 0.001). Control: F_(3,117)_ = 248.85; *p* < 0.001; 10 μM: F_(3,116)_ = 295.41; *p* < 0.001; 25 μM: F_(3,115)_ = 173.26, *p* < 0.001; 50 μM: F_(3,117)_ = 210.87, *p* < 0.001; 100 μM: F_(3,116)_ = 158, *p* < 0.001; 200 μM: F_(3,116)_ = 152.51, *p* < 0.001; 400 μM: F_(3,111)_ = 139.24, *p* < 0.001). Comparison of mean body length (mm) at the same time point in relation to concentration (a for the group of 24 h, b for 48 h, c for 72 h and d for 96 h). The GLM analysis shows: (a) 24 h, F_(1,6)_ = 1.00, *p* = 0.421; (b) 48 h, F_(1,6)_ = 1.469, *p* = 0.190; (c) 72 h, F_(1,6)_ = 3.675, *p* = 0.002; (d) 96 h, F_(1,6)_ = 5.688, *p* < 0.001. Vertical bars represent means and confidence intervals of means. The sample size participating in each analysis is shown by the degrees of freedom (df) following F.

General linear model (GLM) analysis indicated that body length was independently influenced by concentration (F_(6,836)_ = 5.326, *p* < 0.001) and time (F_(3,836)_ = 1331.040, *p* < 0.001) as well as their interaction (cisplatin concentration × time of exposure) (F_(18,836)_ = 2.270, *p* = 0.002). During the studied period (up to 96 h of exposure), the body length of the control embryos increased significantly over time of exposure, with a statistically significant difference observed across time points (F_(3,121)_ = 248.859; *p* < 0.001), consistent with typical larval ontogeny. The mean body length (StDEv) was 3.06 (0.18) mm, 3.50 (0.15) mm, 3.86 (0.16) mm and 4.14 (0.12) mm at 24, 48, 72, and 96 h of exposure respectively. A similar growth pattern was noted for embryos exposed to cisplatin concentrations of 10 to 200 μM cisplatin (Figure 1). The pattern of body length change in relation to cisplatin concentration at the same time period showed that at 24 and 48 h, no statistically significant differences in body length were found. However, at 72 and 96 h, statistically significant differences in body length were found (Figure 1). Overall, the mean body length decreased statistically significantly in relation to concentration. The slope of the line presenting the change in body length in relation to concentration at 96 h was greater than that at 72 h (Figure 1).

**Figure 2:**
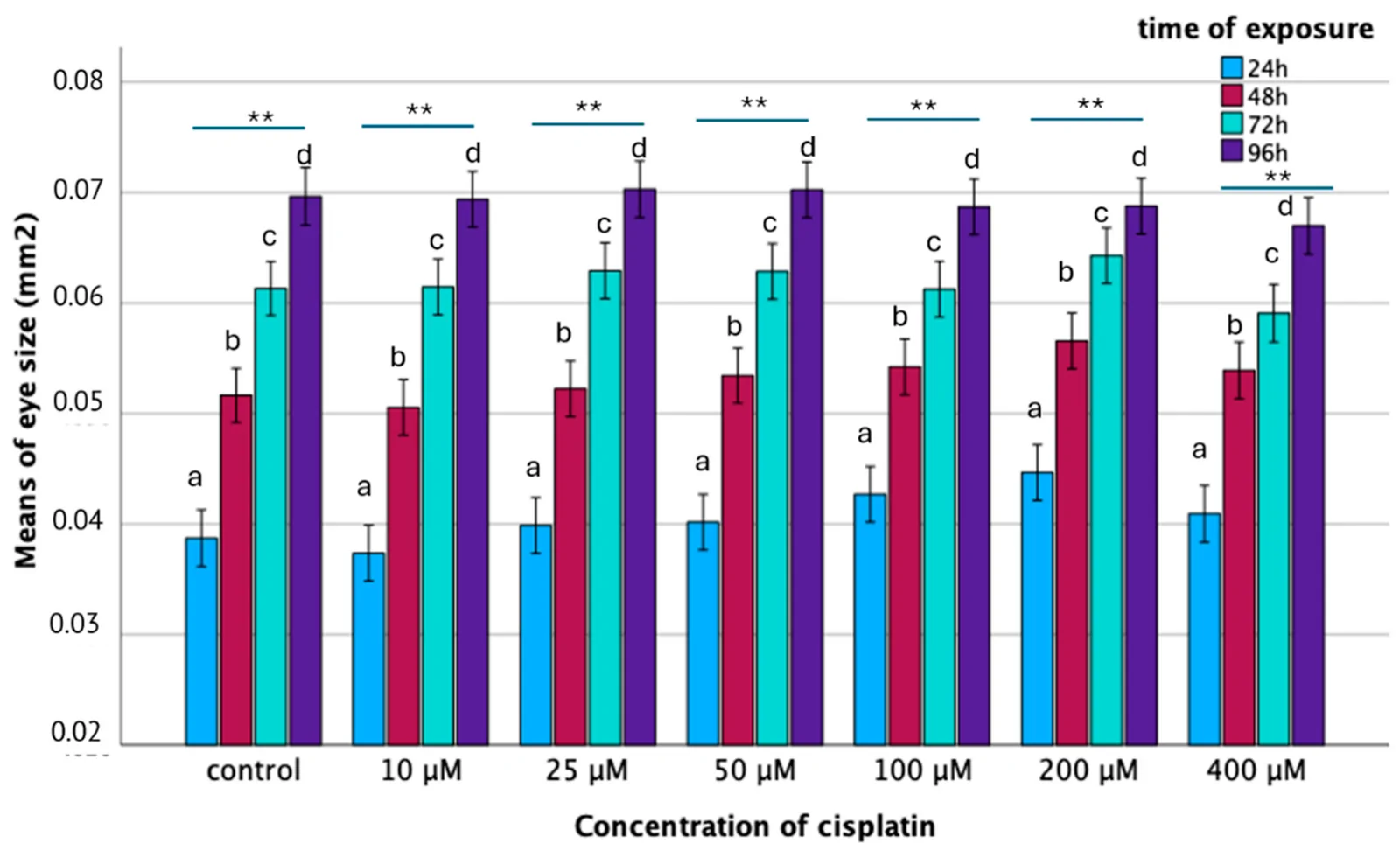
Mean Eye size (mm^2^) of zebrafish embryos exposed to various concentrations of cisplatin per time period (hours of exposure). Horizontal bars above the groups show the comparison within each concentration in relation to different time points. Asterisks denote the level of significance (** indicates *p* < 0.001). Control: F_(3,117)_ = 85.635; *p* < 0.001; 10 μM: F_(3,116)_ = 182.562; *p* < 0.001; 25 μM: F_(3,115)_ = 119.012, *p* < 0.001; 50 μM: F_(3,117)_ = 112.522, *p* < 0.001; 100 μM: F_(3,116)_ = 65.839, *p* < 0.001; 200 μM: F_(3,116)_ = 82.890, *p* < 0.001; 400 μΜ: F_(3,111)_ = 60.496, *p* < 0.001). Comparison of eye size (mm^2^) at the same time point in relation to concentration (a for the group of 24 h, b for 48 h, c for 72 h and d for 96 h). The GLM analysis shows: (a) 24 h, F_(1,6)_ = 4.480, *p* < 0.001; (b) 48 h, F_(1,6)_ = 3.097, *p* = 0.006; (c) 72 h, F_(1,6)_ = 1.375, *p* = 0.226; (d) 96 h, F_(1,6)_ = 0.700, *p* = 0.650. Vertical bars represent means and confidence intervals of means. The sample size participating in each analysis is shown by the degrees of freedom (df) following F.

Cisplatin had a similar effect on the eye surface as on the length. General linear model (GLM) analysis indicated that eye surface area was independently influenced by concentration (F(_6,836)_= 4.237, *p* < 0.001) and time (F_(3,836)_ = 655.566, *p* < 0.001), while there was no significant effect of their interaction (cisplatin concentration × time of exposure) (F_(18,836)_ = 1.483, *p* = 0.089). Ocular development followed a predictable growth curve, with eye surface area expanding progressively from 24 to 96 hpf (*p* < 0.001) at every concentration. While most exposed groups showed no significant deviation from the control (*p* > 0.05), the 200 μM concentration notably disrupted this trend. Embryos in this group exhibited a significantly smaller eye surface area compared to both the control (*p* = 0.004) and the 10 μM and 400 μM cohorts. These data suggest that within the tested range, the 200 μM dosage uniquely impedes ocular expansion (Figure 2).

### 3.4. Heart Rate

**Figure 3:**
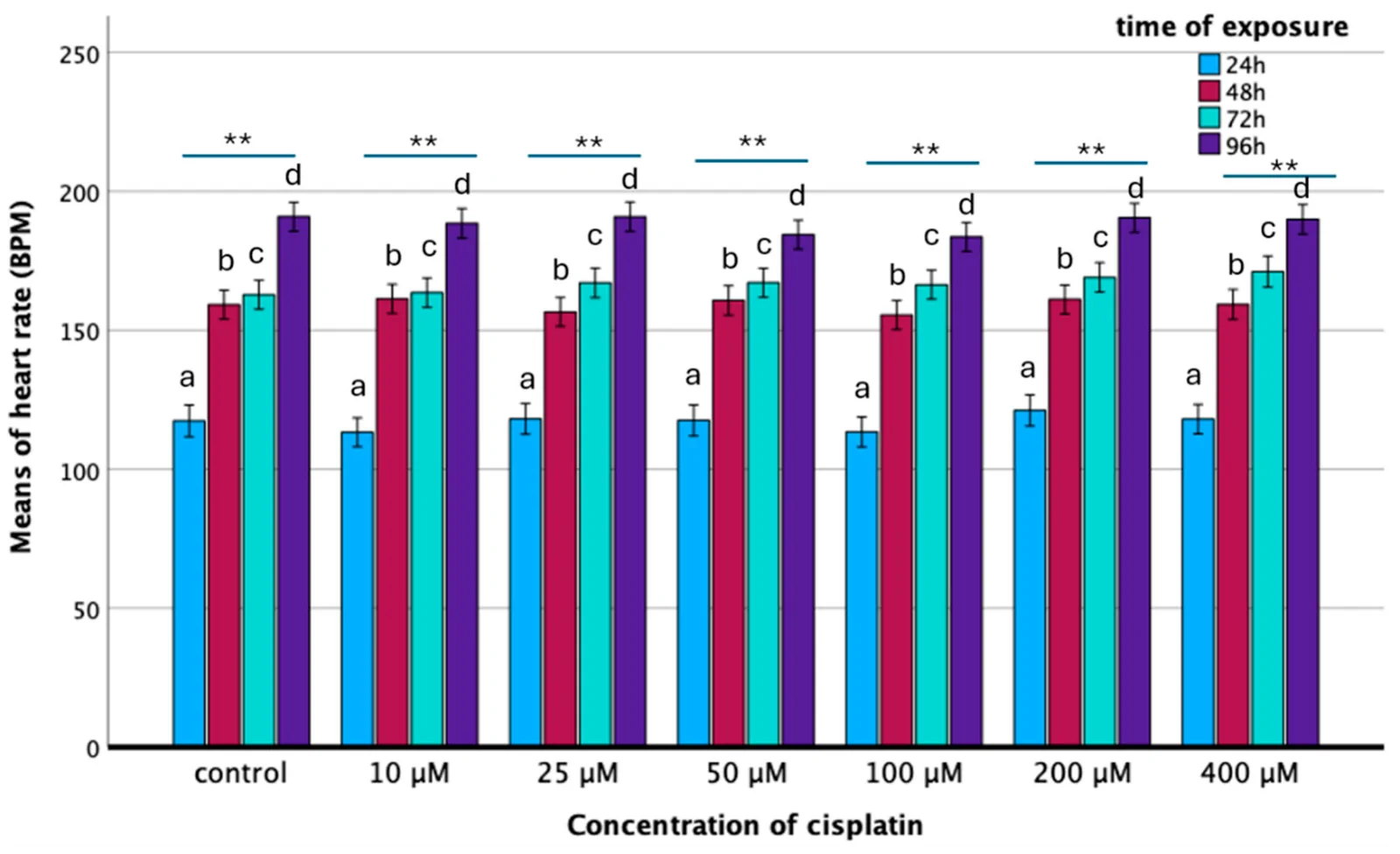
Heart rate (bpm) of zebrafish embryos exposed to various concentrations of cisplatin per time period (hours of exposure). Horizontal bars above the groups show the comparison within each concentration in relation to different time points. Asterisks denote the level of significance (** indicates *p* < 0.001). Control: F_(3,115)_ = 157.502; *p* < 0.001; 10 μM: F_(3,118)_ = 127.098 *p* < 0.001; 25 μΜ: F_(3,115)_ = 122.496, *p* < 0.001; 50 μM: F_(3,115)_ = 101.777, *p* < 0.001; 100 μM: F_(3,118)_ = 131.025, *p* < 0.001; 200 μM: F_(3,116)_ = 106.129, *p* < 0.001; 400 μΜ: F_(3,111)_ = 106.029, *p* < 0.001). Comparison of heart rate (bmp) at the same time point in relation to concentration (a for the group of 24 h, b for 48 h, c for 72 h and d for 96 h). The GLM analysis shows: (a) 24 h, F_(1,6)_ = 0.506, *p* = 0.803; (b) 48 h, F_(1,6)_ = 1.018, *p* = 0.415; (c) 72 h, F_(1,6)_ = 1.141, *p* = 0.05; (d) 96 h, F_(1,6)_ = 1.660, *p* = 0.133. Vertical bars represent means and confidence intervals of means. The sample size participating in each analysis is shown by the degrees of freedom (df) following F.

The heart rate of developing embryos was expressed as the number of beats per minute (bpm) and was investigated in relation to exposure time (hours) and cisplatin concentration. The mean heart rate (stdev) of the non-exposed group was 117.36 (16.778), 159.20 (9.838), 162.80 (12.554), and 190.90 (10.701) (N = 30) at 24, 48, 72, and 96 h of exposure, respectively, exhibiting an increasing trend throughout zebrafish embryonic development. Heart rate (BPM) increased significantly from 24 to 96 hpf (*p* < 0.001), reflecting normal cardiovascular development. In contrast, cisplatin exposure had no broad impact on heart rate. Most comparisons remained non-significant (*p* > 0.05), with an isolated difference observed only between the 100 μM and 200 μM groups. Statistical modeling confirmed that while time was a critical factor (F = 832.018, *p* < 0.001), neither concentration (*p* = 0.072) nor their interaction (*p* = 0.726) significantly altered cardiac frequency (Figure 3).

### 3.5. Behavioral Analysis

#### 3.5.1. Larval Activity

**Figure 4:**
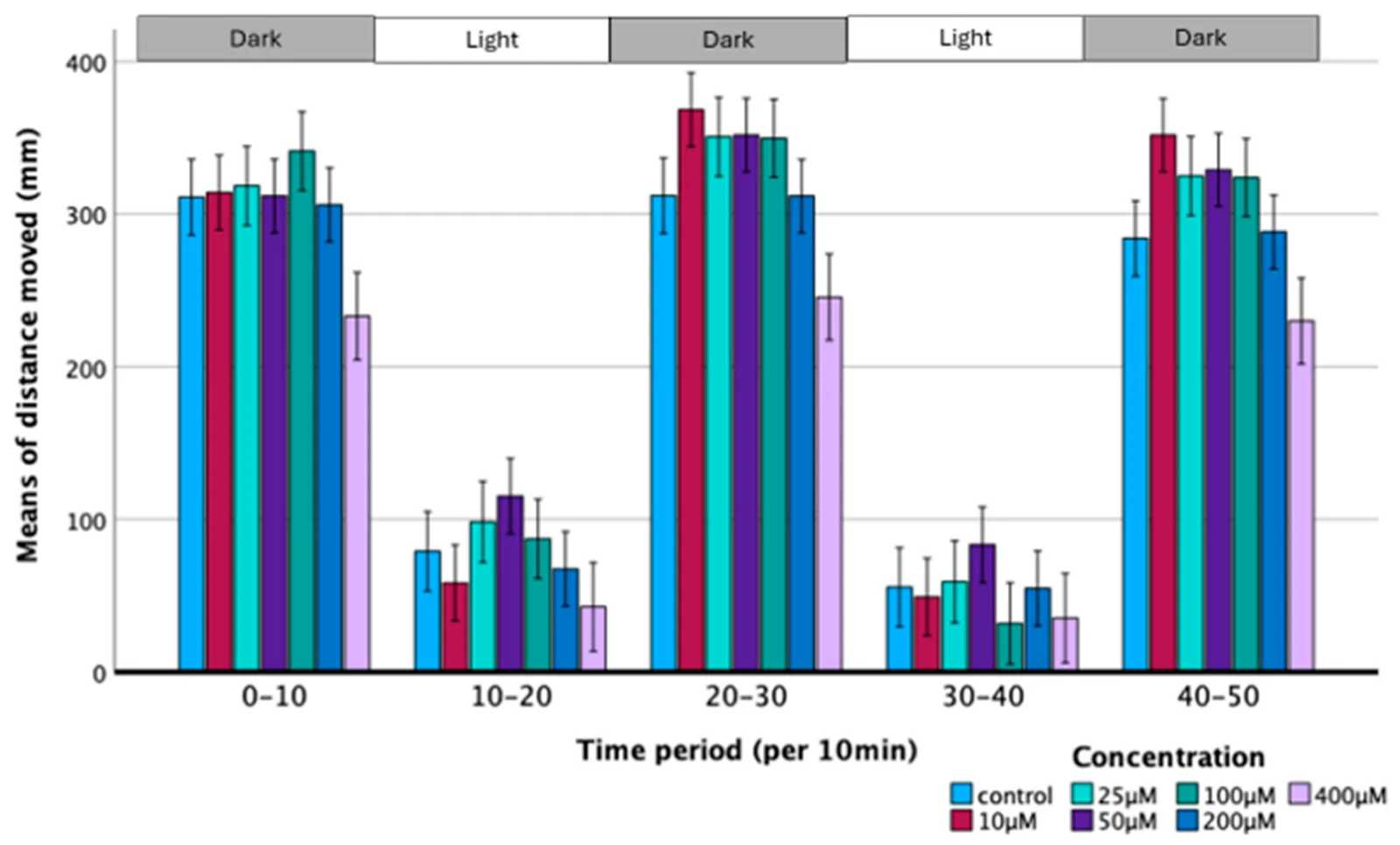
The motor response of zebrafish larvae after 4 days of exposure is strongly impaired by cisplatin. Locomotor activity of control and specimens exposed to 10 μΜ–400 μΜ cisplatin was monitored in a 50-min period of dark/light cycling (0–10 min, dark; 10–20 min, light; 20–30 min, dark; 30–40 min, light and 40–50 min, dark). The distance moved by exposed specimens was quantified at intervals of 2 min and was compared with that of the control. The GLM analysis for cisplatin concentration shows: F_(1,6)_ = 17.643; *p* < 0.001. Bars represent means and confidence intervals of means.

Cisplatin exposure induced concentration-dependent effects in zebrafish larval locomotor behavior, specifically in mean distance movement, compared to the control group. The control group exhibited greater distance movement during the dark phase than in the light phase (Figure 4). Specimens exposed to concentrations between 10 μM and 200 μM of cisplatin exhibited a greater, but not statistically significant, mean distance moved compared to controls. Most notably, embryos exposed to concentrations of 400 μM cisplatin demonstrated a significant reduction in locomotor activity under both light and dark conditions compared to all other groups (hypolocomotion) (Figure 4).

Additionally, a significant difference in activity levels was recorded between the 50 μM and 200 μM groups (*p* < 0.01).

#### 3.5.2. Thigmotaxis Assay

**Figure 5:**
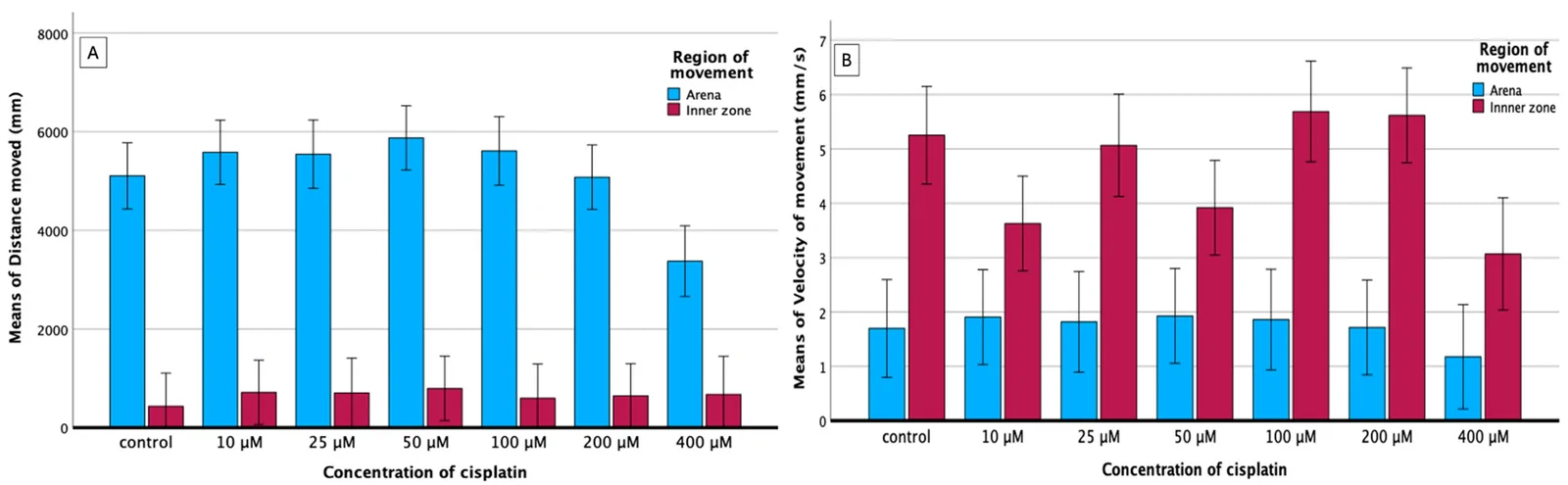
Effect of cisplatin on thigmotaxis in terms of distance moved (**A**) and velocity of movement (**B**) in relation to concentration. Cisplatin concentration significantly affects the region of movement (arena/inner zone) ${(F}_{6,439}=6.188$, p<0.001), as well as the velocity of movement ($F_{6,439}=3.021$, p=0.007). Vertical bars represent means and confidence intervals of means. The sample size participating in each analysis is shown by the degrees of freedom (df) following F.

The GLM analysis revealed a highly significant main effect for spatial zone across all studied behavioral parameters, reflecting the baseline avoidance of the center region characteristic of larval thigmotaxis (Figure 5).

There was a significant main effect of cisplatin concentration ($F_{6,439}=6.188$, p<0.001) and a significant interaction effect between concentration and arena zone ($F_{6,439}=6.188$, p<0.001). While larvae across lower concentrations (0−200 μM) spent minimal time in the center (control inner mean: 227.12±190.63 s), exposure to 400 μM cisplatin significantly increased the total time spent in the inner zone (715.98±782.53 s; Tukey HSD/Bonferroni post-hoc p<0.001 vs. control).

Total distance traveled differed significantly across arena zones ($F_{1,439}=593.167$, p<0.001) and exhibited a significant concentration × zone interaction ($F_{6,439}=2.490$, p=0.022). Larvae exposed to 400 μM cisplatin exhibited a decrease in overall arena distance moved (3374.17±3557.27 mm) compared to 50 μM-treated larvae (5872.42±2202.24 mm, p=0.013).

Velocity of movement differed significantly between the arena and inner zones ($F_{1,439}=134.525$, p<0.001) with a significant concentration effect ($F_{6,439}=3.021$, p=0.007). Mean velocity in the 400 μM group was significantly lower (2.05±2.07 mm/s) compared to the 100 μM group (3.77±3.19 mm/s, p=0.008) and 200 μM group (3.67±4.47 mm/s, p=0.013).

#### 3.5.3. Touch-Evoked Motor Response (TMR) and Vibrational Startle Response (VSR)

**Figure 6:**
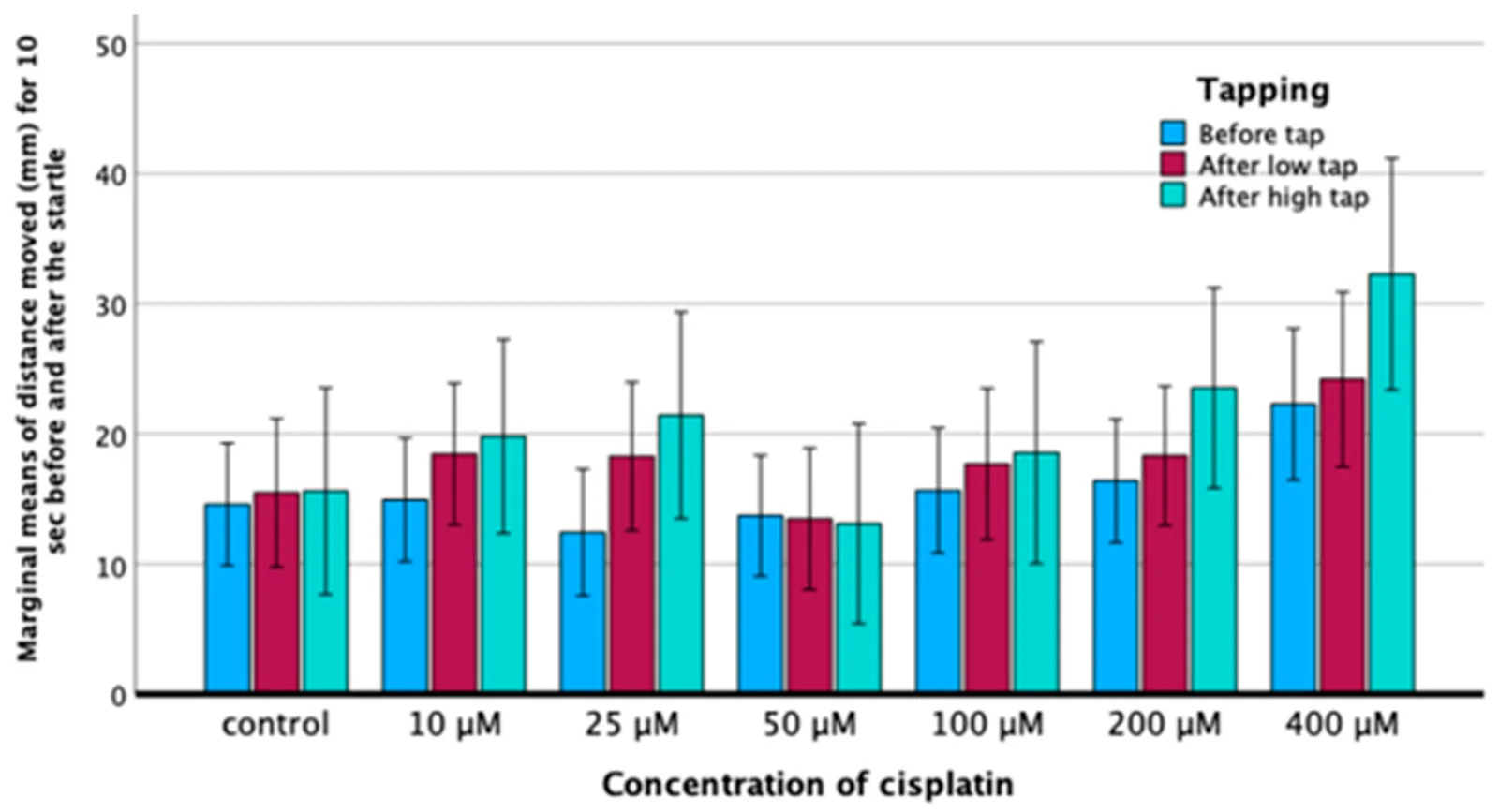
Distance moved (cm) by zebrafish larvae for 10 s before and after a stimulus delivery (Low intensity (1×) and High intensity (4×)) in relation to increasing concentration of cisplatin (0–400 μM). Distance moved was significantly increased according to concentration ($F_{\left(6,567\right)}=4.115$, p<0.001) and stimulus intensity ($F_{\left(2,567\right)}=3.895$, p=0.021). Error bars represent means and confidence intervals of means. The sample size participating in each analysis is shown by the degrees of freedom (df) following F.

GLM analysis demonstrated a statistically significant main effect for both cisplatin concentration ($F_{\left(6,567\right)}=4.115$, p<0.001) and stimulus intensity ($F_{\left(2,567\right)}=3.895$, p=0.021) on total distance moved. However, the interaction between concentration and stimulus intensity was not statistically significant ($F_{\left(12,567\right)}=0.429$, p=0.952). Post-hoc analyses indicated that larvae exposed to the highest concentration (400 μM) exhibited significantly greater distance moved compared to the control group and lower concentration groups (p<0.05). Furthermore, locomotor activity was significantly increased following the high-intensity stimulus (high tap) relative to baseline activity prior to the tap (p=0.019) (Figure 6).

### 3.6. Application of the Embryo-Adapted Protocol to Cell-Cycle Analysis in Whole Embryos

To assess the practical ability of the embryo-adapted workflow to capture a cellular response within the exposure model, we performed flow cytometric DNA-content analysis on whole zebrafish embryos. This approach was used to assess whether exposure to increasing cisplatin concentrations induces redistribution among the major cell-cycle phases of individual embryos. This approach provides insights into possible effects on proliferative activity and cell-cycle regulation, corroborating the utility of a rapid cellular readout complementary to the organism-level endpoints described above.

**Figure 7:**
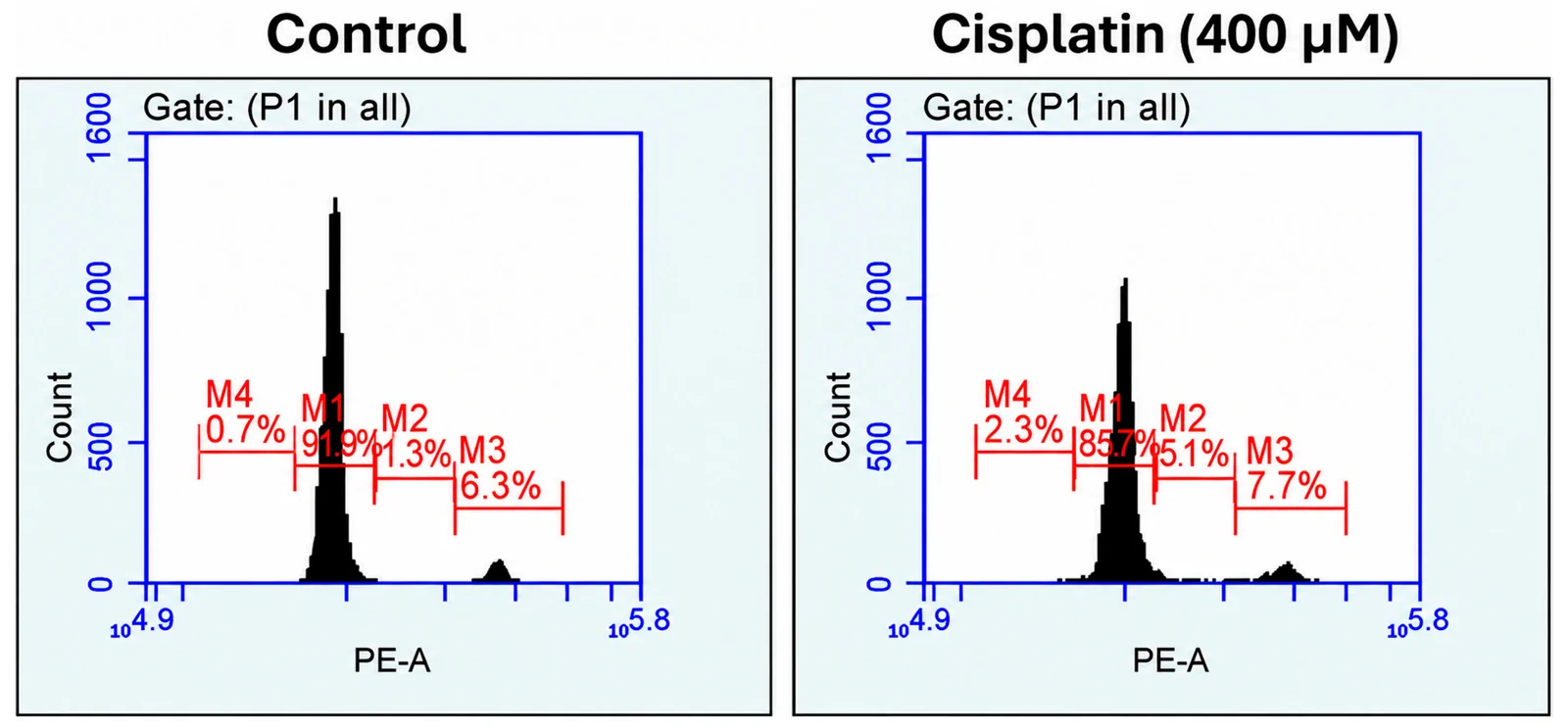
Representative DNA-content histograms obtained by flow cytometric analysis of cells derived from an untreated control zebrafish embryo and an embryo exposed to 400 μM cisplatin. Cell-cycle distribution was determined by measuring propidium iodide (PI) fluorescence intensity (PE-A), which reflects cellular DNA content. The main population (M1) corresponds to cells in G0/G1 phase with diploid DNA content, while M2 represents cells in S phase, characterized by intermediate DNA content during DNA synthesis. M3 corresponds to cells in G2/M phase, which contain increased DNA content, and M4 represents the subG1 population, typically associated with fragmented DNA and apoptotic cells.

A representative DNA-content histogram is shown in Figure 7. Based on propidium iodide fluorescence intensity, cell populations were assigned to the subG1, G0/G1, S, and G2/M phases according to their DNA content. This analysis allowed the quantification of the relative proportion of cells in each phase and enabled comparison between control and cisplatin-treated groups.

**Figure 8:**
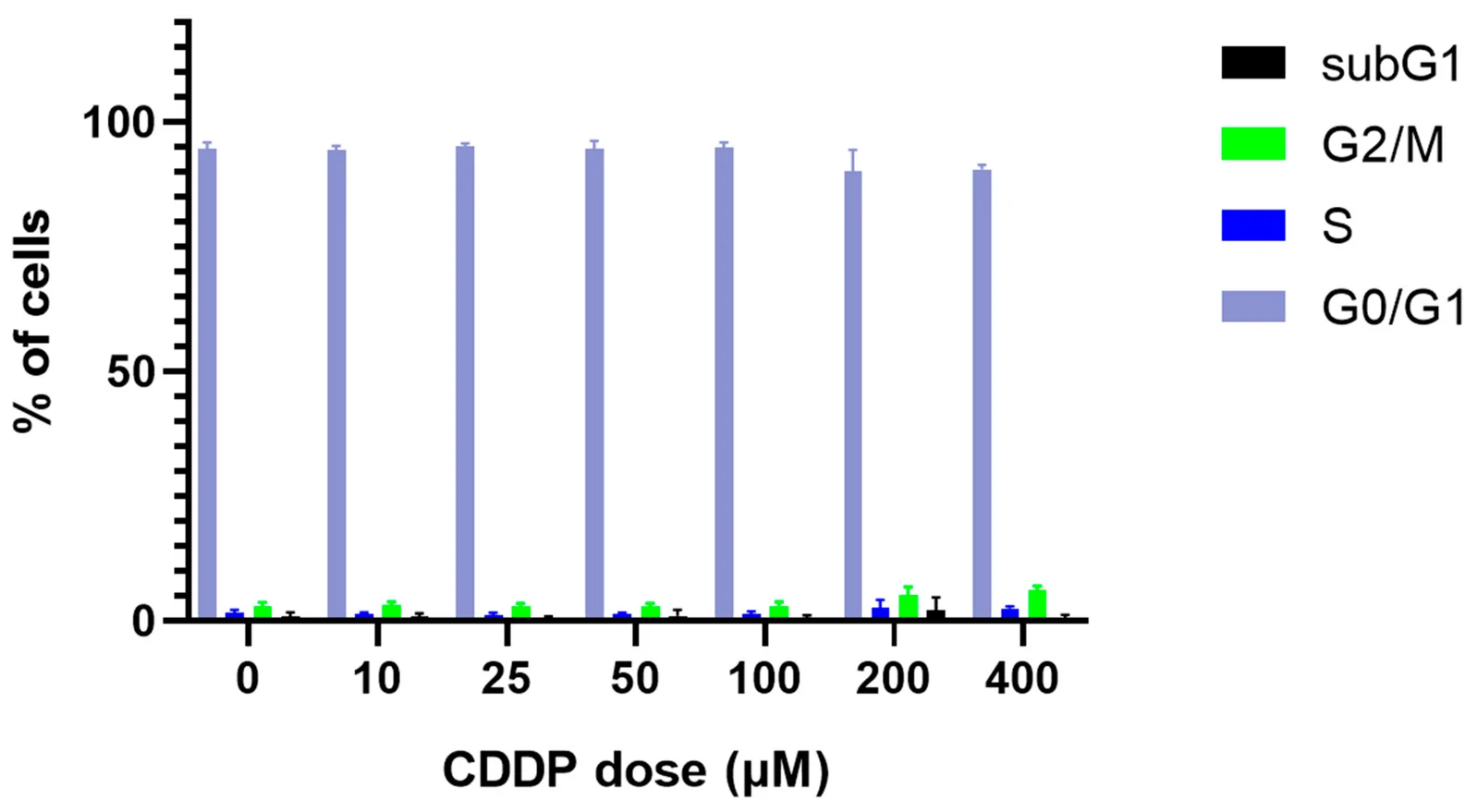
Effect of cisplatin (CDDP) on cell-cycle distribution in zebrafish embryos. Embryos exposed to increasing concentrations of CDDP (0–400 μM) were analyzed by flow cytometry, and the percentages of cells in G0/G1, S, G2/M, and subG1 phases were determined. The graph was generated from pooled data of the experimental runs. Data are presented as mean ± SD from three independently performed experimental series. In each experimental series, three individual embryos were analyzed separately per condition, with each embryo constituting one flow cytometry sample and no pooling performed.

Flow cytometric analysis of DNA content demonstrated that cisplatin induced changes in the cell-cycle profile of zebrafish embryos, and this effect was confirmed in an independent biological replicate. The results are presented in Figure 8.

The majority of cells remained in the G0/G1 phase in control embryos and in embryos exposed to the lower cisplatin concentrations (10–100 μM), indicating that low-dose treatment did not markedly affect the overall cell-cycle profile. In contrast, exposure to the higher concentrations (200–400 μM) was associated with a reduction in the proportion of cells in G0/G1 together with an increase in the G2/M fraction, consistent with an alteration in normal cell-cycle progression. A modest increase in the S-phase population was also observed in some of the higher-dose groups, while the subG1 fraction remained generally low but showed a slight increase at the highest concentrations. Overall, the combined data indicate that cisplatin affects cell-cycle progression in zebrafish embryos in a dose-related manner, with the clearest and most consistent changes occurring at the highest concentrations tested.

Post hoc analysis showed that the most consistent changes were observed at the highest cisplatin concentration. Compared with control embryos, the G0/G1 fraction was significantly reduced at 400 μM (90.58 ± 0.87% vs. 94.70 ± 1.23%, adjusted *p* = 0.002), while the G2/M fraction was significantly increased at 400 μM (6.22 ± 0.87% vs. 2.83 ± 0.96%, adjusted *p* = 0.001). At 200 μM, the same trend was observed, with reduced G0/G1 (90.12 ± 4.35%) and increased G2/M (5.13 ± 1.70%), although these differences did not remain significant after correction for multiple comparisons. The S-phase fraction showed a modest increase at high dose (2.53 ± 0.42% at 400 μM vs. 1.68 ± 0.52% in controls), but this change did not remain significant after multiple-comparison adjustment. No statistically significant differences were detected in the subG1 fraction. Collectively, these findings indicate that high-dose cisplatin primarily alters cell-cycle distribution by decreasing the proportion of cells in G0/G1 and increasing the proportion in G2/M.

## 4. Discussion

In this study, we combined standard zebrafish developmental-toxicity design with an embryo-adapted rapid DNA-content flow cytometry workflow. This design allowed organism-level morphometric, cardiac, and neurobehavioral findings to be interpreted alongside a cellular measure of cell-cycle distribution. Across the 10–400 μM concentration range and 96-h exposure period, cisplatin produced distinct developmental and behavioral effects, while the flow cytometric readout identified cell-cycle redistribution at the highest concentration. Three main conclusions arise from the current study. First, cisplatin affects body length and eye surface in a concentration- and time-dependent manner, with effects becoming obvious only after 48 h of exposure. Second, cardiac function, assessed by heart rate, was remarkably preserved across all tested concentrations, in contrast to the marked growth impairment observed for other endpoints. Third, the highest concentration tested (400 μM) produced a complex and, in places, apparently paradoxical behavioral response, combining spontaneous hypolocomotion, reduced thigmotaxis, and an increased locomotor response to touch and vibrational stimuli.

### 4.1. Growth Impairment: Body Length and Eye Surface Area

The progressive, concentration-dependent reduction in body length, which became statistically significant only at 72 and 96 h post-exposure, is consistent with the delayed onset of overt developmental toxicity typically reported for platinum-based compounds in early vertebrate life stages. According to Siddik (2003) cisplatin exerts its principal cytotoxic action through the formation of platinum-DNA adducts, predominantly 1,2-intrastrand cross-links, which trigger the DNA-damage response and activate ATR/p53/p73-dependent signaling; when the resulting lesions cannot be adequately repaired, affected cells undergo cell-cycle arrest and, ultimately, apoptosis [20]. Because early zebrafish development depends on rapid, near-synchronous rounds of cell proliferation, tissues with the highest mitotic turnover—including the somatic musculature and axial skeleton that determine body length—would be expected to be disproportionately sensitive to an antiproliferative, DNA-crosslinking agent, which is consistent with the comparatively late but then pronounced growth deficit observed here. A parallel mechanism involves cisplatin-induced generation of reactive oxygen species and depletion of cellular antioxidant capacity (glutathione, thioredoxin), which compounds DNA damage and independently promotes apoptotic and inflammatory signalling [21], this oxidative component has been repeatedly implicated as a driver of growth retardation, pericardial and yolk-sac abnormalities, and reduced hatching in other zebrafish developmental toxicity studies, even for structurally unrelated xenobiotics, underscoring oxidative stress as a convergent downstream mechanism of many teratogenic and growth-suppressing chemicals in this model.

Our results are also in general agreement with prior zebrafish-specific work on cisplatin. Hung et al. (2019) reported that cisplatin exposure reduced body length in zebrafish embryos at concentrations from 100 μM upward, and that this growth deficit co-occurred with impaired ionocyte and hair-cell function and increasing whole-body platinum accumulation [22]. The concentration range at which a significant decline in body length was observed, with the effect becoming most pronounced at 200–400 μM by 96 h, is broadly consistent with this prior threshold, despite differences in exposure protocol, continuous immersion from 24 hpf onward versus the dechorionation timing and staging used in earlier reports. This convergence across independent studies strengthens the inference that impaired somatic growth is a robust and reproducible endpoint of cisplatin developmental toxicity in this species.

Unlike body length, which declined in an approximately monotonic, concentration-dependent manner, eye growth was unaffected across most of the tested range and was significantly reduced only at the highest concentration (400 μM This non-monotonic dose-response pattern is observed in developmental studies and may reflect several non-mutually exclusive explanations. This could be attributed to the competing processes operating at different concentrations: at 400 μM, the overall suppression of somatic growth and locomotor activity may proportionally preserve, or cover changes in, relative ocular dimensions, whereas at 200 μM a more selective disruption of optic vesicle proliferation or vascularization becomes apparent before broader systemic toxicity dominates the phenotype. Alternatively, the finding may reflect the statistical reality of multiple concentration-by-time comparisons, in which an isolated 200 μM effect could represent a true but narrow window of ocular vulnerability rather than a generalizable feature of the dose–response curve. Given that ocular development depends heavily on tightly regulated neuroepithelial proliferation and apoptosis, and that platinum-DNA adducts preferentially affect actively dividing tissue, a transient period of heightened sensitivity in the optic primordium cannot be excluded and would be a productive target for follow-up work using proliferation and apoptosis markers restricted to the eye.

### 4.2. Cardiac Frequency: A Comparatively Insensitive Endpoint

In marked contrast to the growth endpoints, heart rate was almost entirely unaffected by cisplatin exposure across the full concentration range. This is a noteworthy finding given that dose-limiting cisplatin toxicity in the clinical setting is not restricted to nephro-, oto-, and neurotoxicity but can also include cardiovascular disturbances, most often secondary to electrolyte derangement such as hypomagnesemia [21], and that the larval zebrafish heart rate assay reliably detects dose-dependent bradycardia or tachycardia for many other cardioactive xenobiotics. Several considerations may account for the relative sparing of cardiac frequency in our study. First, heart rate is widely regarded as a comparatively insensitive and non-specific endpoint of cardiotoxicity in the zebrafish embryo model: it can remain within normal limits even when structural cardiac parameters (chamber morphology, looping, contractility, stroke volume) are already compromised, because simple rate measurements do not capture subtler electrophysiological or morphological derangements. Second, sinoatrial pacemaking in the early larval zebrafish heart may be relatively resistant to acute cisplatin exposure at these concentrations and exposure durations compared with the slowly cumulative, multi-cycle exposure that underlies clinical cisplatin cardiotoxicity in patients. Third, because heart rate continued to increase normally with developmental time across all treatment groups, it is possible that overt cardiotoxic thresholds for cisplatin lie above the highest concentration tested here or would only become apparent with additional structural or functional readouts, such as stroke volume, ejection fraction, or fractional shortening, which were not assessed in the present study.

### 4.3. Locomotor Behavior and Thigmotaxis: Dissociation Between Spontaneous Activity and Anxiety-like State

The behavioral data revealed a striking concentration-dependent dissociation. At low to intermediate concentrations (10–200 μM), larvae tended toward greater, non-significant locomotor activity relative to controls, whereas larvae exposed to the highest concentration (400 μM) displayed pronounced hypolocomotion across both light and dark phases (Figure 4). Sub-threshold hyperactivity followed by high-dose hypoactivity is a recurring pattern in zebrafish behavioral toxicology and is often interpreted as reflecting a transition from a mild stress- or stimulant-like response at sublethal concentrations to overt malaise, neuromuscular impairment, or sickness behavior as the toxic burden increases. Because cisplatin is neurotoxic to peripheral sensory and, to a lesser extent, motor pathways in mammals, chemotherapy-induced peripheral neuropathy in humans can include motor weakness, gait disturbance, and reduced reflexes in addition to its more prominent sensory component [23]. It is expected that the hypolocomotion observed at 400 μM reflects an analogous, general impairment of neuromuscular or sensorimotor integration in the zebrafish larvae, superimposed on the systemic growth deficit already documented at this concentration, rather than a purely CNS-mediated sedative effect.

The thigmotaxis findings add a further layer of interpretive complexity. Thigmotaxis, the tendency of larvae to remain near the peripheral wall of an arena rather than exploring the center, is a well-validated index of an anxiety-like state in larval zebrafish, sensitive to anxiolytic and anxiogenic pharmacological manipulation in a manner that parallels open field behavior in rodents [24]. Under our conditions, larvae exposed to 400 μM cisplatin spent significantly more time in the central (inner) zone than did controls or lower-concentration groups, a pattern that, taken at face value, would suggest an anxiolytic-like or reduced defensive state. However, this same concentration group also travelled a shorter total distance and moved at a lower velocity than intermediate concentration groups, raising the important possibility that the apparent reduction in thigmotaxis is at least partly a locomotor confound rather than a genuine anxiolytic phenotype: severely hypoactive larvae may simply fail to actively patrol the peripheral zone, drifting toward or remaining in the centre by default rather than through active exploration. This ambiguity is a well-recognized limitation of thigmotaxis assays in models with confounding motor impairment, and it argues for caution in ascribing a purely affective interpretation to the 400 μM phenotypes. Disentangling a genuine reduction in anxiety-like state from a locomotor artefact would require normalizing zone preference to total distance travelled, or combining thigmotaxis with a pharmacological probe (e.g., co-exposure to a locomotor stimulant) to determine whether exploratory capacity, rather than exploratory drive, is the limiting factor.

### 4.4. Sensorimotor Responsiveness: Touch-Evoked and Vibrational Startle Responses

Probably the most counterintuitive result of the behavioral battery was that larvae exposed to the highest cisplatin concentration (400 μM), despite showing hypolocomotion in the open-swimming assays, exhibited a significantly greater distance moved than controls following touch and vibrational (tap) stimuli, with locomotor activity further increasing after the high-intensity stimulus. This apparent contradiction reduced spontaneous activity alongside an exaggerated evoked response is not unprecedented for cisplatin and may be understood considering its well-characterized effects on peripheral mechanosensory structures. In zebrafish, the lateral-line neuromasts contain mechanosensory hair cells that are structurally, molecularly, and functionally homologous to the hair cells of the mammalian cochlea and are a validated in vivo model for cisplatin ototoxicity [25]. Critically, cisplatin uptake into these hair cells, and the resulting cell death, has been shown to depend on functional mechanotransduction itself: pharmacological or genetic disruption of the mechanotransduction channel blocks both drug entry and hair-cell death [26], implying a tight, dose-dependent coupling between mechanosensory activity and cisplatin-induced peripheral sensory injury.

Two non-exclusive interpretations follow from this literature. On one hand, sub-lethal hair-cell or afferent damage at high cisplatin concentrations could plausibly lower the threshold for stimulus-evoked escape responses or produce a form of mechanosensory hypersensitivity analogous to the allodynia and hyperalgesia that characterize chemotherapy-induced peripheral neuropathy in mammals, where sensitized nociceptive and mechanoreceptive afferents produce exaggerated behavioral responses to normally innocuous or moderate stimuli [23]. On the other hand, the touch-evoked and vibrational startle circuits recruit fast, largely reflexive escape pathways (mediated substantially by Mauthner-cell-initiated C-start responses) that are anatomically and physiologically distinct from the slower, more voluntary swimming circuits engaged during spontaneous light–dark locomotion; it is therefore conceivable that cisplatin differentially spares or even sensitises the former while suppressing the latter, rather than uniformly depressing all motor output. Either interpretation is consistent with our observation that the interaction between concentration and stimulus intensity was not statistically significant, suggesting that the exaggerated evoked response at 400 μM reflects a shift in overall responsiveness rather than a stimulus-specific sensitisation. Given that hair-cell loss in the zebrafish lateral line is dose-dependent and can occur within hours of cisplatin exposure [24], direct visualization of neuromast hair-cell integrity (e.g., using vital dyes such as FM1-43 or DASPEI) alongside the behavioral assays would help clarify whether peripheral sensory pathology, central sensitization, or a combination of both underlies this dissociation.

### 4.5. Integration: A Concentration-Dependent Shift from Proliferative to Sensorimotor Toxicity

Viewed together, the results of this study appear to trace a concentration-dependent shift in the dominant mechanism of cisplatin toxicity. At low to intermediate concentrations (10–100 μM), effects were subtle or absent for most concentrations, consistent with the comparatively low sensitivity of larval zebrafish to the antiproliferative, DNA-damaging actions of cisplatin relative to actively dividing mammalian tissues such as the bone marrow or gastrointestinal epithelium. At 200 μM, a more selective vulnerability emerged, manifesting principally as impaired ocular growth and altered thigmotactic behavior, which may reflect tissue- and circuit-specific sensitivities that are not yet fully resolved. At 400 μM, the phenotype broadened into a systemic toxicity syndrome encompassing growth restriction, spontaneous hypolocomotion, reduced peripheral thigmotaxis (with the locomotor caveats noted above), and exaggerated stimulus-evoked responses, a combination that is most parsimoniously explained by the convergence of the antiproliferative/genotoxic mechanism described above with a peripheral sensorimotor component analogous to cisplatin ototoxicity and neurotoxicity in mammals. This pattern reinforces the broader principle, well established in the clinical cisplatin literature, that nephrotoxicity, ototoxicity, and neurotoxicity, though mechanistically related through shared oxidative stress and DNA damage pathways, do not necessarily co-occur at the same dose or timescale, and can present with distinct thresholds and trajectories even within a single organism [20].

The flow cytometric findings complement these organism-level observations. At the highest cisplatin concentration, the reduction in the G0/G1 population together with the increase in G2/M indicates altered cell-cycle progression and coincides with the concentration at which the most pronounced growth and behavioral effects were observed. Although the present data do not establish a direct causal relationship between cell-cycle redistribution and the observed phenotypes, they demonstrate that rapid DNA-content analysis can provide an additional cellular-level readout alongside conventional developmental and behavioral endpoints.

### 4.6. Methodological Significance of the Embryo-Adapted Rapid DNA-Content Workflow

Beyond the toxicological findings, the methodological contribution of this study is the transfer of the rapid Ioannina DNA-content workflow from fresh tissue specimens to whole zebrafish embryos. The embryo-adapted procedure combines mechanical dissociation, filtration, brief PI/RNase staining, and immediate flow cytometric acquisition, providing a cellular readout within the same experimental framework used for developmental and behavioral phenotyping. Importantly, the workflow operates at the single-embryo level, with one embryo per sample and no pooling. In this proof-of-application, the method detected the clearest and most reproducible cell-cycle shift at 400 μM cisplatin, with a reduction in G0/G1 and an increase in G2/M. The workflow is intended to complement, rather than replace, conventional zebrafish endpoints. Further work should establish minimum embryo input, cell recovery, inter-operator reproducibility, and agreement with independent proliferation or DNA-damage markers before broader standardization.

### 4.7. Limitations of the Current Study

Several limitations should be acknowledged. The flow cytometric component of the study was designed as a proof-of-application of the embryo-adapted workflow and was performed in three independent experimental series, with three individual embryos analyzed separately per condition in each series. Larger experimental datasets will be useful to further establish the reproducibility and biological variability of the single-embryo approach. In addition, even though heart-rate and behavioral measurements represent functional endpoints, some behavioral parameters, particularly thigmotaxis, may be influenced by concurrent alterations in locomotor activity. Therefore, the present findings should primarily be considered as a methodological demonstration accompanied by exploratory biological observations.

## 5. Conclusions

This study demonstrates the feasibility of integrating single-embryo rapid DNA-content flow cytometry with the standard developmental and behavioral assessment in zebrafish. The embryo-adapted workflow detected cisplatin-related cell-cycle redistribution at higher concentrations and provided a complementary cellular readout alongside organism-level endpoints. These findings support the further use and validation of this rapid, non-pooled approach in zebrafish developmental and toxicological studies.

## Data Availability

The original contributions presented in this study are included in the article. Further inquiries can be directed to the corresponding authors.
